# Alprazolam role in the analgesic effect of ibuprofen on postendodontic pain

**Published:** 2014

**Authors:** Mahmoud Baradaran, Mahmoud Reza Hamidi, Mohammad Reza Moghimi Firoozabad, Sohrab Kazemi, Manouchehr Ashrafpour, Ali Akbar Moghadamnia

**Affiliations:** 1 Department of Pharmacology, Babol University of Medical Sciences, Babol, Iran.; 2 Department of Endodontics, Babol University of Medical Sciences, Babol, Iran.; 3 Cellular & Molecular Biology Research Center, Babol University of Medical Sciences, Babol, Iran.

**Keywords:** Alprazolam, Ibuprofen, Pain, Postendodontic pain, Benzodiazepines

## Abstract

***Background: ***Postendodontic pain (PEP) has always been a major problem for patients and dentists and NSAIDs are being used to relieve PEP and it is supposed that some benzodiazepines may potentiate facilitate the analgesic effects of the NSAIDs. This study was conducted to evaluate the effect of alprazolam on the analgesic effect of ibuprofen in PEP treatment.

***Methods:*** This randomized double-blind clinical trial was conducted on 45 patients aged 20-45 years who were subjected of root canal treatment. A written informed consent was obtained from each patient. The subjects were randomly divided into three groups; placebo, ibuprofen (400 mg) and alprazolam (0.5) mg + ibuprofen (400 mg). The intensity of pain was recorded using visual analog scale (VAS) at 4, 6, 12, 24, 48 and 72 hours after drug administration.

***Results:*** Of the participants, twenty six (57.8%) were males and 19 patients (42.2%) were females. Four hours after starting treatment, the VAS scores in the placebo and ibuprofen -treated groups were significantly higher than ibuprofen and alprazolam+ibuprofen groups (4.93±1.16, 3.67±1.88 and 2.67±1.11, respectively, p<0.0001). The VAS scores in alprazolam + ibuprofen group (2.33±1.05) were significantly lower at 6 hours after treatment when compared to the other groups (Ibuprofen: 3.00±1.36 and placebo: 3.08±1.74, P=0.002). This decrease in VAS score sustained to 12 hours after the start of alprazolam + ibuprofen treatment when compared to ibuprofen or placebo receiving group alone (p<0.003). The average pain score in female patients who received alprazolam + ibuprofen was significantly lower than males at 12 hours (1.3±0.6 *v.s* 2.14±0.9, P=0.002) and 24 hours after treatment (0.88±0.6 v.s 1.86±0.9, P=0.003).

***Conclusion: ***According to the results, it can conclude that alprazolam may enhance the analgesic efficacy of ibuprofen in postendodontic pain.

Although, endodontic therapy is done without pain in the entire duration of the work, but still the pain may be experienced after treatment. Based on the patient’s variability, postendodontic pain (PEP) could vary from mild to severe (1). PEP may be triggered by the stimulation of pain pathways following the release of chemical pain mediators in damaged tissues (2). Pathophysiology of pain is complicated and peripheral and central pathways are involved in the control of pain. There are both physiological and psychological aspects and failure to control pain which can lead to the patient’s failure to cooperate and follow-up the treatment. Effective control of the pain is an important topic in dental procedures (3). 

Usually the easiest way to treating and relieving the pain of dental procedures is through the use of non-steroidal anti-inflammatory drugs (NSAIDs) which act by inhibition of the cyclooxygenase pathway, the synthesis of prostaglandins and thromboxane is reduced and among NSAIDs is ibuprofen, which is more commonly used (-). The first generation drugs in this category are non-selective inhibitors of the cyclooxygenase (COX I, II). The enzyme COX I is found throughout the body and plays an important role in protecting the gastrointestinal mucosa, normal kidney function and platelets (9). 

COX II is involved in the production of prostaglandins that are pain mediators and the inhibition of this enzyme could prevent the production of these types of prostaglandins (10). Ibuprofen and mefenamic acid are examples of the most common drugs in this category (11). 

The combined analgesics with the different mechanisms of action may exert synergistic effect and can improve the management of postoperative pain in dental procedures (12). After treatment, the irritation of peripheral receptors and subsequent inflammation is prevented (13). 

Some NSAIDs like ibuprofen have advantages as compared to others, including the lower incidence of gastrointestinal ulcers (14), less damage to the kidneys and decreasing platelets (15). Ibuprofen has been used for its analgesic effect for PEP (16). 

Benzodiazepines are among the other drugs that can be used as adjuvant drugs in times of pain that may enhance the analgesic effects of NSAIDs (17, 18). Various studies have shown that alprazolam and other benzodiazepines act as positive modifiers and potent activators of GABA and GABA_A_ receptors in the CNS (-). 

GABA receptor agonists showed significant analgesic properties in the animal models of pain studies. Thus, by targeting these receptors involved in the regulation of pain threshold, some new drugs may be introduced for pain controlling goals (22, 23). Therefore, the use of NSAIDs in combination with other drugs such as benzodiazepines may increase their efficiency.

However, successful pain control is still one of the most important issues in dentistry and nowadays, several medical analgesic protocols are proposed for PEP treatment (12). The synergistic effect of simultaneous use of sedatives and analgesics has been considered. This combination therapy may help to reduce the dose of analgesic drugs as well as decrease the risk of adverse drug reactions. Some studies suggest that low doses of benzodiazepines such as alprazolam can enhance the analgesic properties of NSAIDs and/or morphine compared to their administration alone (24). Clinically, benzodiazepines are used widely in combination with opioids to increase their analgesic effects. However, few results on the effectiveness of benzodiazepines used as an adjunctive treatment of pain control had been reported (-). 

Ibuprofen has been commonly used in dental pain and benzodiazepines may be used for sedation during and after dental painful procedures. Based on the mentioned above, the present study has been designed and implemented to determine the possibly analgesia - enhancing effect of alprazolam on ibuprofen in postendodontic pain.

## Methods

This randomized double-blind clinical trial was conducted on 45 patients 20-45 (30.4±6.9) years old who referred to the Department of Endodontics, the Dental School of Babol University of Medical Sciences (Babol, Iran). The proposal was approved by the University Committee of Medical Ethics and all patients signed a written informed consent after explaining to them about the investigation and type of drugs. This project was registered in IRCT (Iranian registry of clinical trials) data bank with registration number: IRCT201312219271N4.

The inclusion criteria were: no systemic diseases, no drug allergy to NSAIDs and benzodiazepines, not receiving any other sedatives and analgesics, no gastrointestinal problems, and no pregnancy or breast feeding during the study. Three participants were excluded from the investigation due to failure to provide pain relief. The participants should not receive analgesics at least 4 hours before starting the investigation. The initial pain score reported by patients was in a range of moderate to severe. The pain should only be reported in the molar tooth. 

This means that the person had irreversible pulpitis (i.e. the tooth was not necrotic and dental pain in the studied patient was caused only by molar tooth and the other teeth had no pain). The subjects were randomly divided into three groups (n=15 in each group). Lidocaine 2%+1/80000 epinephrine (Daroupakhsh Co. Tehran, Iran), gutta percha (Aryadent, Tehran, Iran), AH26 sealer (Dentsply, Germany), gelatin capsules (Daroupakhsh, Tehran, Iran), sucrose (Merck Germany), ibuprofen 400 mg (Arya Co. Tehran, Iran), alprazolam 0.5 mg (Abidi Co., Tehran, Iran) and acetaminophen 325 mg (Daroupakhsh, Tehran, Iran) were purchased.

The endodontic treatment was done for the patients in two sessions. In the first session of treatment, access cavity was prepared using master apical file (MAF). During this step, the prepared canals were washed with saline solution and cleaned and dried using sterile cotton. All the canals were dressed in this step. 

For local anesthesia, lidocaine 2% containing epinephrine 1:80000 was used. At the end of the first session of treatment, the patients in each group have randomly received one of the three treatments; placebo, ibuprofen 400 mg and alprazolam 0.5 mg with ibuprofen 400 mg. All drugs were encapsulated encoded and administered as single dose. The patients were called, completed the data sheet including the personal information and pain score assessment. They were asked to record their pain scores at 4, 6, 12, 24, 48 and 72 hours after receiving drugs based on a visual analog scale (VAS) method (27). 

Meanwhile, a package containing two tablets of acetaminophen 325 mg as rescue dose was given to each participant to take if the pain persists. The patients were asked to record the rescue dose in the form once used. In the second session of treatment, the canals were filled with gutta percha and AH26 sealer and after dressing the tooth, the patient was referred for a crown restoration.

Data were extracted from the patient’s data sheets and were presented as mean±standard deviation (SD) and analyzed using One-Way ANOVA post hoc Tukey and repeated measures tests. Any difference between data was considered statistically significant at p-value less than 0.05.

## Results

All 45 patients completed the study. Twenty-six (57.8%) of the participants were males and 19 (42.2%) were females. The mean and standard deviation of age of patients in placebo, ibuprofen and ibuprofen+alprazolam groups were 30.66±7.26, 31.4±64.4 and 29.13±7.18 years, respectively. There was no significant difference between the gender of the patients in the 3 groups (P=0.54).

Table 1 compares the VAS scores in three treatment groups and shows the significance level in the results of the different treatments. The average pain score in placebo- treated control group was greater than ibuprofen (p<0.05) and ibuprofen+alprazolam (p<0.0001) receiving groups. Six hours after treatment, the average VAS score in ibuprofen + alprazolam group was significantly lower than ibuprofen group (2.33±1.05 *vs* 3.00±1.36, P=0.018) and placebo group obviously (P=0.018). Twelve hours after the treatment, the average VAS score in ibuprofen + alprazolam group was significantly lower than the placebo group (1.60±0.91 *vs *3.60±2.29, p<0.001). The female patients in the ibuprofen + alprazolam group reported an average VAS score less than the male patients 12 hours after treatment (1.12±0.64, *vs* 1.85±0.89, P=0.002). The comparison of VAS score at 24, 48 and 72 hours between ibuprofen and ibuprofen + alprazolam groups showed no significant differences (p>0.5). Twenty-four hours after the treatment of females in ibuprofen + alprazolam group, showed a VAS score lower than the males (0.87±0.64 *vs*1.85±0.89, P=003). The time dependent variation in VAS scores showed an intra-group significant differences in three treatment groups (p<0.0001).

**Figure 1:**
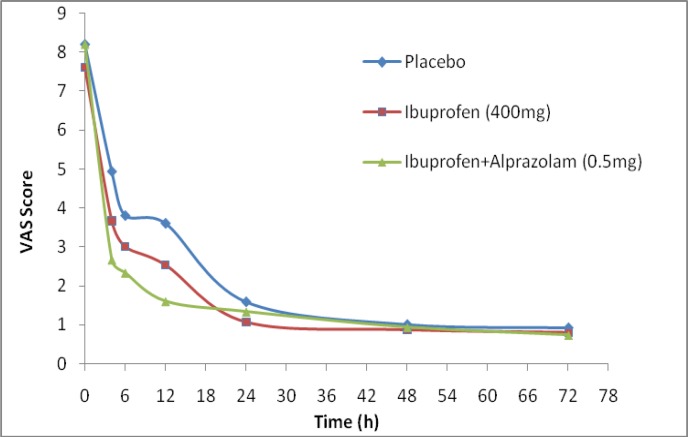
Comparison of average VAS scores in post endodontic pain (PEP) of three treatment groups, placebo, ibuprofen 400mg and ibuprofen 400mg+ alprazolam 0.5mg (the number of patients in each group was fifteen and, at 4 hr; p<0.05, at 6 hr; p=0.018, at 12 hr; p=0.003)

Figure 1. shows the changes in VAS scores before and after treatment through pain assessment in patients of three treatment groups.

**Table1:** Mean (±SD) of VAS score in patients with post endodontic pain (PEP) in placebo, ibuprofen and ibuprofen + alprazolam treatment groups

**Group**	**Before**	**4H**	**6H**	**12H**	**24H**	**48H**	**72H**
^a^Placebo	8.20 (1.01)	4.93 (1.16)	3.80 (1.74)	3.60 (2.29)	1.58 (0.90)	1.00 (1.04)	0.92 (1.00)
^b^Ibuprofen (400mg)	7.60 (0.99)	3.67 (1.88)	3.00 (1.36)	2.53 (1.06)	1.07 (1.22)	0.87 (1.25)	0.80 (1.15)
^c^Ibuprofen(400mg)+ Alprazolam (0.5mg)	8.20 (1.26)	2.67 (1.11)	2.33 (1.05)	1.60 (0.91)	1.33 (0.90)	0.93 (0.88)	0.73 (0.59)
pvalue	NS	a, b: 0.05 a, c: 0.0001	a, c: 0.018	a, c: 0.003	NS	NS	NS

## Discussion

The results show that alprazolam can increase the effect of ibuprofen on alleviating PEP compared to ibuprofen alone. This result is inconsistent with previously reported investigations (25, 28). Some authors reported the different results of the role of benzodiazepines interaction (24, -). It has been reported that NSAIDs may induce analgesic effect via inhibition of cyclooxygenase in the spinal cord (24). Ibuprofen in comparison to some older NSAIDs, has lower side effects but effective control on PEP (31). For this reason, in this investigation using alprazolam to obtain enhanced analgesic effect was considered. It is shown that ibuprofen can alleviate the migraine headache within 2 hours post treatment (32), which is consistent with the present study but some investigations have shown different results (33, 34). In the present study, the average VAS score at 4 and 6 hours after treatment in ibuprofen + alprazolam group was lower than the score of ibuprofen group. The drug can take affect within 4-6 hours after ingestion and can reach to the peak blood level within 10-12 hours post treatment. This may be due to the improving effect of alprazolam on the effect of ibuprofen. Benzodiazepines can stimulate GABA_A _receptors in dorsal horn of spinal cord and this mechanism may enhance the analgesic effects of NSAIDs (35). The GABA receptor agonists are clinically effective in the treatment of pain, especially when combined with other analgesics. Benzodiazepines reduces the pain intensity by reducing the pain-induced anxiety, insomnia and muscle spasms. The other possible mechanism of action of alprazolam can be stimulating in the release of endogenous opioids, such as enkephalins in CNS areas involved in pain processing (27, 36). The role of GABA_A_ receptors in spinal nociceptive pathways is well established (37). Enhancing the analgesic effect of ibuprofen by alprazolam may be attributed to its effects on the GABA_A_ receptors. Many studies have shown PEP during the first 24 to 48 hours after treatment (38, 39). Previous studies showed that women have more pain after root canal therapy (40). But some studies have also stated that there is no significant difference between males and females (41) that is inconsistent with the results of our study. In the present study, the average VAS score in females receiving ibuprofen + alprazolam was less than the males that was consistent with the results of previous studies (40, 42). In this respect, our previous study also showed the increased analgesic effect of ibuprofen via alprazolam. It was concluded that adding alprazolam to the treatment might be more effective than ibuprofen alone in dysmenorrhea treating symptoms (26).

 In the present investigation, the patients receiving ibuprofen and ibuprofen + alprazolam have significantly lower pain score than the placebo and ibuprofen receiving subjects at 4, 6, and 12 hours after the treatment, But in one study, no significant difference has been reported between placebo and ibuprofen in PEP 12 hours after treatment (42), that is inconsistent with our findings. Some patients of the ibuprofen group reported nausea and vomiting more than the patients of ibuprofen + alprazolam group; this effect might be due to the anti-nausea effect of benzodiazepines such as alprazolam (43). Self-medication other than analgesics during the investigation and overestimation to declare pain score may be important limitations of this study. 

In conclusion, although the exact mechanism of action of alprazolam effect in enhancing analgesia induced by NSAIDs remains to be elucidated, it can be concluded that alprazolam can enhance the analgesic effect of ibuprofen in postendodontic pain and also can reduce the side effects of ibuprofen. 

Authors’ contributions 

MB: research coordinator, study design; MRH: clinical part supervision, study design and patients management; MRM: patient handling, data collection and executive affairs; MA: references double checking and reviewing the text of the manuscript; SK: registering study in IRCT, preparing reference library and writing coordination, literature searching; AAM: data handling and statistical interpretation, manuscript correction. All authors read and approved the final manuscript.

## Notes

**Funding:** This investigation (thesis No:9235516) was done as a DDS thesis project of Mohammad Reza Moghimi, and it was financially supported by the Research Affairs Division of Babol University of Medical Sciences.
